# Efficient dynamic channel assignment through laser chaos: a multiuser parallel processing learning algorithm

**DOI:** 10.1038/s41598-023-28282-z

**Published:** 2023-01-24

**Authors:** Zengjing Chen, Lu Wang, Chengzhi Xing

**Affiliations:** Zhongtai Securities Institute for Financial Studies, Shandong University, Jinan, 250100 China

**Keywords:** Electrical and electronic engineering, Fibre optics and optical communications, Information technology

## Abstract

As laser chaos has been proven to be a robust tool to solve the multi-armed bandit (MAB) problem, this study investigates the problem of multiuser dynamic channel assignment using laser chaos in cognitive radio networks with *K*-orthogonal channels and *M* secondary users. A novel dynamic channel assignment algorithm with laser chaos series for multiple users, named parallel processing learning with laser chaos (PPL-LC) algorithm, is proposed to efficiently address two main objectives: stable channel assignment and fuzzy stable channel assignment. The latter objective accounts for the realistic scenario where users have fuzzy preferences and do not necessarily pursue the best preference. The PPL-LC algorithm uses the randomness properties of laser chaos to learn the assignment of channels to multiple users without any limitations on the number of channels, which has not been considered in existing laser chaos algorithms. Moreover, the PPL-LC is equipped with parallel processing channel selections, resulting in higher throughput and stronger adaptability with environmental changes over time than comparison algorithms, such as distributed stable strategy learning and coordinated stable marriage MAB algorithms. Finally, numerical examples are presented to demonstrate the performance of the PPL-LC algorithm.

## Introduction

Since cognitive radio networks (CRNs) were proposed^1^, multiuser dynamic channel assignment has been a significant focus. Advances in terminology suggest improvements in the performance of CRNs based on resource utilization and other factors. As primary users have priority over secondary users in spectrum utilization, secondary users should be aware of primary users’ behaviors. For efficient resource utilization, corresponding issues, such as which channel primary users choose and what time to sense, should be considered by secondary users. Existing spectrum allocation and sharing approaches are discussed in detail^2^. Zhao and Sadler^3^ reviewed various models with dynamic spectrum access. Using the Gale–Shapley stable matching algorithm, the stable marriage problem of multiuser dynamic channel assignment was first solved under the assumption that the expected rates are known and a distributed opportunistic CSMA protocol that solves the problem was proposed^4^. This approach has been shown to have a range of advantages and has received significant attention recently.

The combination of the multi-armed bandit (MAB) problem and cognitive radio access was initially studied with independent channels^5^. Channel selection is considered as a type of MAB problem; that is, the amount of information passed through a channel is considered as the gain obtained by a robot arm. Lai, Jiang, and Poor^6^ discussed the solution to the cognitive media access problem and considered various scenarios using the upper confidence bound (UCB) algorithm to explore the optimal choice of numerous users and channels in this problem. Further, they proved that the UCB algorithm achieves an expected sum of regrets of near-$O(\log T)$. Avner and Mannor^7^ proposed the concept of stable marriage configuration (SMC) and proposed the coordinated stable marriage MAB (CSM-MAB) algorithm to reach the SMC state. The CSM-MAB algorithm requires communication and exchange of channels between different users, but it does not give the approximate order achieved by the regret. Bistritz and Leshem^8^ proposed The game of thrones (GoT) algorithm, which does not require user communication, and proved that it achieves an expected sum of regrets of near-$O(\log ^{2}T)$. Gafni and Cohen^9^ introduced a fully distributed algorithm—distributed stable strategy learning (DSSL)—to solve a multiuser channel assignment problem and achieved a stable state through a large number of exchanges between users with the upper boundary of the regret given in the theoretical proof.

Several recent observations on laser chaos motivate its application to dynamic channel assignment^10^. In physics, chaos has always been closely related to randomness and is extremely sensitive to the initial parameter values. The chaotic oscillation of a laser signal level is generated by the injection of delayed light into the cavity of a diode laser emitter, which is accomplished by directing the output of the semiconductor laser through a beam splitter toward a laser reflector. The resulting chaotic oscillation of the laser signal level can be harnessed for various applications, such as secure communication, chaos-based sensing, and chaos-based control. Based on this, random numbers are obtained by the fast sampling of the signal level^11^. Random numbers generated by laser chaos are generally in the GHz or higher level, indicating the high quality of these random numbers. They seem natural and have passed the National Institute of Standards and Technology (NIST) random number test. Considering these advantages, research has been conducted on potential applications of laser chaos. Naruse et al.^12^ proposed a MAB algorithm using chaotically oscillating waveforms. Subsequently, the algorithm was further improved to consider the order correct probability of MAB^13^. The major work inspiring this study is the use of a laser chaos algorithm to solve the problem of dynamic channel selection for a single user^14^.

Although laser chaos has been proven to be effective in the aforementioned literature, there still exists some engaging questions that need to be addressed. One of these questions is whether laser chaos can perform well in CRNs with multiple users. In other words, it is worth investigating whether it is possible to achieve higher throughput by laser chaos to attain a suitable stable configuration. Focusing on stable configuration, it should be noted that the preferences of different users in the real world are not consistently precise, which prompts contemplation on how to dynamically select channels in CRNs based on the fuzzy preferences of users. Therefore, this study investigates dynamic channel selection in CRNs using laser chaos series based on a proper stable aim.

Based on the above starting points, a novel dynamic channel assignment algorithm with laser chaos series for multiple users, named parallel processing learning with laser chaos (PPL-LC) algorithm, is proposed to enhance throughput and improve operational efficiency. To increase the verisimilitude of the proposed model, consider a CRN comprising multiple users and multiple channels. The probability of a given channel may differ among the various users, adding a layer of complexity and realism to the model. The stable channel assignment (SCA) has aroused widespread interest. As a result, a new definition for the stable arrangement of channels based on users’ fuzzy preferences, called fuzzy stable channel assignment (FSCA), has been proposed. This described the reality of situations where users have their recognition interval^15,16^, and all channels in this interval are equally possible to be chosen. Therefore, the channel selection problem is studied to achieve SCA and FSCA separately. The PPL-LC algorithm includes two stages with the users’ channel preference updating process, and the parallel channel exchange process. As MAB problems require a balance between exploration and exploitation, laser chaos provides an efficient tool for sufficient exploration. Based on this, in the process of updating users’ preferences, the PPL-LC algorithm applies laser chaos to achieve equilibrium for multiple users. During the channel exchange process, a parallel method that selected users simultaneously perform the procedure is employed.

In the following, detailed comparisons with related results are provided. First, the previous work only proposed a stable state similar to SMC, whereas this paper considers FSCA state. The performance of the proposed algorithm is evaluated by targeting these two states. Based on the experiment, the PPL-LC algorithm has a higher throughput than the CSM-MAB and DSSL algorithms. Second, the restriction of existing laser chaos algorithms based on channels (arms) that are integer powers of 2 is eliminated and the algorithm is generalized to support an arbitrary number of channels. Third, to the best of our knowledge, this is the first time laser chaos is applied to dynamic channel selection with multiple users. In addition, to attain higher throughput and perform more processes, this work introduces a parallel channel exchange processing to reach two target stable states. The principles of this processing approach are presented in detail below.

## Principles

### Characteristics of CRNs

This paper considers the following scenario to investigate the CRN problem. In an information communication network system with *K* channels and *N* users, it is assumed that each user can only use one channel and that each channel allows only a single user to transmit information. If two or more users occupy the same channel simultaneously, the information passed through the channel becomes null. Users can communicate with each other to exchange channels. Without loss of generality, the assumption $K \le N$ is taken in this study. Otherwise, the number of users transmitting information concurrently can be restricted through the use of split time to satisfy the condition $K \le N$.

When user *n* selects the channel *k* for information transmission, two random outcomes may occur: complete transmission or rejection. Complete transmission signifies that all information passes through channel *k*, whereas rejection denotes the state in which no information passes through channel *k*. For convenience, the symbols used in this paper are defined as follows.

- $\mathcal {U}=\{1,2,\ldots , N\}$ indicates the set of users and $\mathcal {C}=\{1,2,\ldots , K\}$ is the set of channels.
- $X_{n,k}$ indicates the binary random variable of whether user *n* can completely transmit 1 bit of information using channel *k*, which is consistent regardless of time step.
- $\mu _{n,k}$ indicates the expectation of $X_{n,k}$, i.e., $\mathbb {E}[X_{n,k}]$.
- $\pi _{n,t}$ indicates the channel assignment of the user *n* at time *t*.
- $T_{t}^{n,k}$ indicates the number of times the user *n* selects the channel *k* before time *t*.
- $R_{t}^{n,k}$ indicates the total benefit of the user *n* choosing channel *k* before time *t*.
- $\hat{\mu }_{t}^{n,k}$ indicates the average benefit of the user *n* choosing channel *k* before time *t*, i.e,
  $$\begin{aligned} \hat{\mu }_{t}^{n,k} = \frac{R_{t}^{n,k}}{T_{t}^{n,k}}. \end{aligned}$$

**Figure 1:**
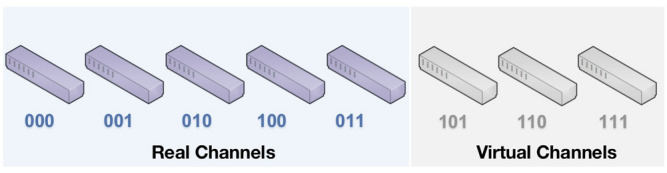
Extension of number of channel by adding virtual channels with five channels as an example.

As mentioned in Introduction, the existing laser chaos algorithms usually assume that the number of arms (channels) satisfies the condition $K= 2^{M}$ for $M \in \mathbb {N}^{*}$. However, real situations rarely satisfy this assumption. To be coincident with reality, this study removes this restriction such that *K* can take any number. For any number of channels $K>1$, a positive integer *M* can be found such that $2^M<K \le 2^{M+1}$. A sequence of binary codes $\{S_{1}S_{2} \ldots S_{M+1}\}$ can be used to represent each channel $k\in \mathcal {C}$ with $S_{i} \in \{0,1\}$ for $i = 1,\ldots ,M+1$. The laser chaos algorithm uses $2^{M+1}$ as the number of channels and sets $\mu _{n,k_{a}}$ to zero for all $k_{a}$ among the last $2^{M+1} -N$ channels with $n = 1,\ldots ,N$. For example, consider the case where $K=5$ and all channels can be represented as a set of binary codes $\{000,001,\ldots ,111\}$. The last three channels $\{101,110,111\}$ are designated as virtual channels (Fig. 1).

For channel distributions, a general model is considered where reward differs for various users in the same channel. In other words, the probability that a channel exhibits a fully transmitted state depends on the users. This assumption fits the situation, in complex electromagnetic environments, that the channel environment changes according to distinct users. Each user has *B* bits information waiting to be delivered. According to the additivity of distribution, the amount of information that user *n* transmits from the *B* bits information through channel *k* is $BX_{n,k}$. If two or more users choose the same channel simultaneously, the collision results in $X_{n,k}=0$. An allocation strategy $\pi _n$ for user *n* is defined as a vector of time $\{\pi _{n,t},n\in {\mathcal {U}}, t=1,2,\ldots \}$. Therefore, the total amount of information $R_t$ transmitted by all users at time *t* is defined as

$$\begin{aligned} R_t = B\sum \limits _{t}\sum \limits _{n} {\mathbb {E}}[X_{n,\pi _{n,t}}]. \end{aligned}$$

Unlike the single-user model, optimal decisions of multiuser dynamic channel selection are determined by target state setting. This study consider two matching objectives: SCA^4^, which is widespread in existing literature, and FSCA, which takes vague choice preferences into account. The following provides detailed descriptions of these two definitions.

#### Definition 1

SCA indicates the strategy mapping $\mathcal {M}_S$ from $\mathcal {U}$ to $\mathcal {C}$ that for any $n \in \mathcal {U}$ and $k \in \mathcal {C}$ satisfying $\mathcal {M}_S(n)\ne k$, if $\mu _{n,k}-\mu _{n,\mathcal {M}_S(n)}>0$, then there exists some user $n_0\in \mathcal {U}$ such that $\mathcal {M}_S(n_0)=k$ and $\mu _{n_0,k}-\mu _{n,k}>0$.

#### Definition 2

FSCA indicates the strategy mapping $\mathcal {M}_F$ from $\mathcal {U}$ to $\mathcal {C}$ that for any $n \in \mathcal {U}$ and $k \in \mathcal {C}$ satisfying $\mathcal {M}_F(n)\ne k$, if $|\mu _{n,k}-\mu _{n,\mathcal {M}_F(n)}|\le \delta$, then there exists some user $n_0\in \mathcal {U}$ such that $\mathcal {M}_F(n_0)=k$, $|\mu _{n_0,\mathcal {M}_F(n)}-\mu _{n_0,k}| >\delta$ or $|\mu _{n_0,k}-\mu _{n,k}|> \delta$.

**Figure 2:**
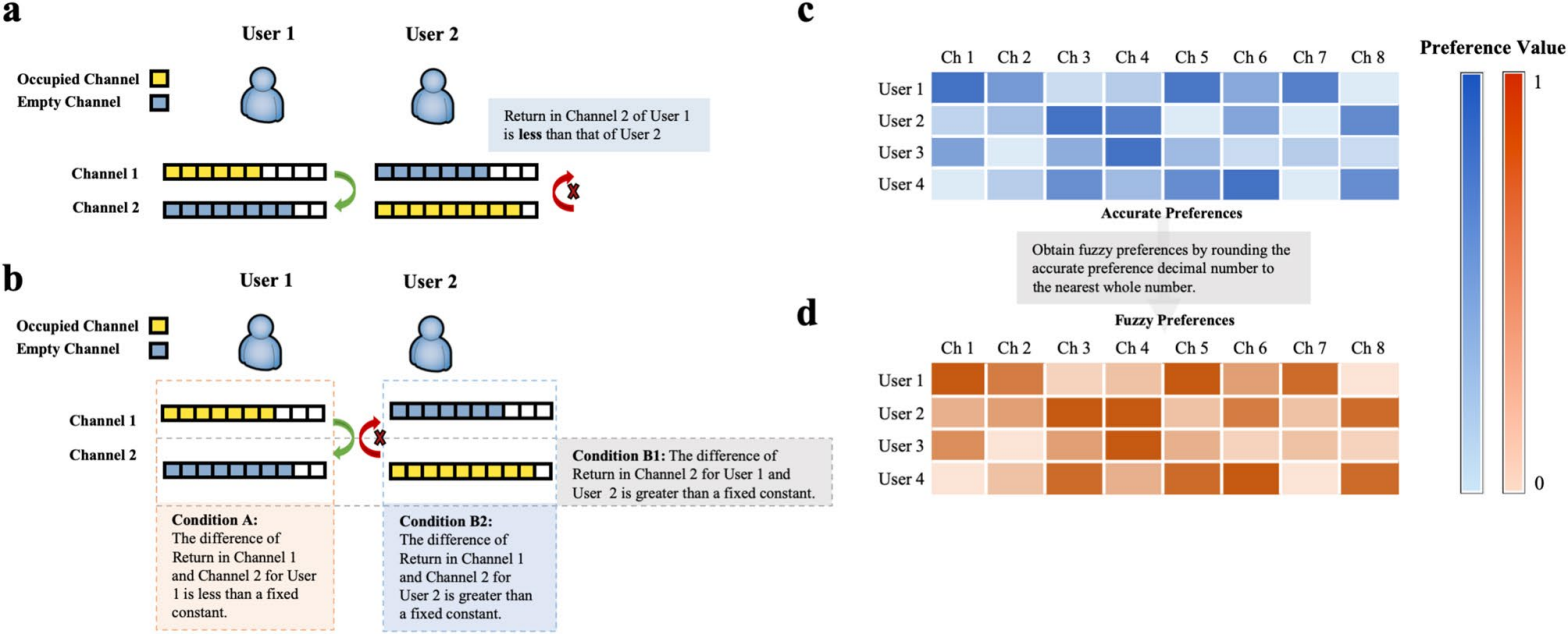
SCA and FSCA instructions for two users and eleven channels. (**a**) The SCA state where user 1 is willing to exchange, and user 2 is reluctant to exchange. (**b**) The SCA state where Conditions A and B1 are satisfied but Condition B2 is not. (**c**) The users’ preferences with specific cognition ranging from 0 to 1. (**d**) The users’ preferences with vague cognition obtained from **(c)**.

The difference between SCA (Fig. 2a) and FSCA (Fig. 2b) is users’ subjective choice, which reflects that FSCA is a weaker match than SCA. From the definitions, it is noticed that a strategy is FSCA if it satisfies Conditions A and B, including the two alternatives Condition B1 and B2 (Fig. 2b). In other words, FSCA will be reached if one of Condition B is satisfied. A more intuitive representation of fuzzy preferences is presented in Fig. 2c,d. If the user has a very clear acknowledgment of a channel, the good or bad preference can be seen through the shade of blue. The darker the color, the more satisfied the user is. It can be seen that, in this case, the color of blue changes a lot, and many level divisions exist (Fig. 2c). With the same data in Fig. 2c, the user’s preferences are blurred and orange has less color change and less grading (Fig. 2d). In the next subsection, a brief overview of the laser chaos algorithm is presented.

### Laser chaos algorithm

**Figure 3:**
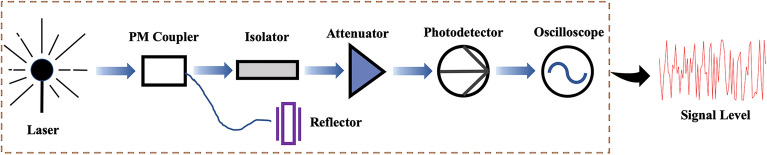
The generation process of laser chaos where a semiconductor laser emits laser light through the beam splitter to a laser reflector, and the delayed light injects into a diode laser emitter cavity.

In previous research, the laser chaos algorithm was experimentally proven to have a correct higher-order probability compared with other algorithms^12^. A correct higher-order probability helps the selection preference to be close to the true channel gain order, which is vital for the subsequent channel exchange. This is also the main reason the laser chaos algorithm is used in this study. As shown in Fig. 3, the semiconductor laser emits laser light through the beam splitter to the laser reflector, and the delayed light injects into the diode laser emitter cavity. Laser chaos is a result of delayed laser feedback, converted to an electrical signal by a photodetector and illustrated by an oscilloscope. Through ultrafast frequency sampling, high-quality random numbers are generated.

Previous studies used the laser chaos algorithm to find the optimal arm (channel) in MAB problems, whereas this study only uses the laser chaos algorithm to update selection preference instead of channel selection. Updating a user’s preference with the laser chaos algorithm is divided into three steps as follows.

**Step 1: Select the channel according to the random number generated by laser chaos.**

Throughout the previous discussions, any channel number can be converted into $2^{M}$. Thus, use $\{S_{1}S_{2} \ldots S_{M}\}$ to represent all channels. The channel can be selected by *M*-times comparisons between the signal levels at different times and the corresponding threshold. Denote $s(t_{i})$ as the signal level value generated by the laser chaos at time $t_{i}$ and $t_{1}$ as the initial time point of the first channel selection. $T^{H}_{i,S_{1}\ldots S_{i-1}}$ indicates the threshold of selecting $S_{i}$ on the basis that $S_{1}\ldots S_{i-1}$ has been determined.

If $s(t_{i})$ is no less than $T^{H}_{i,S_{1}\ldots S_{i-1}}$, set $S_{i}$ to 1. Otherwise, $S_{i}$ is set to 0 for $i = 1,\ldots ,M$. In this round of channel selection, $\Delta _{S}$ is the time interval to select $S_{i}$ for $i = 1,\ldots , M$ in sequence. Naturally, the initial time point is $t_{1}+\Delta _{S}$ in the next round of channel assignment and a similar procedure can be performed in the subsequence.

**Step 2: Send a message and receive feedback.**

Send the message through the channel $k' = S'_{1}\ldots S'_{M}$ selected in the previous step and obtain the feedback. The feedback value is equivalent to the observation of random variables. Then, record $T_{t}^{n,k'}$ and $R_{t}^{n,k'}$ and update $\hat{\mu }_{t}^{n,k'}$ of channel $k^{'}$ at time slot *t*.

**Step 3: Adjust the threshold according to the feedback obtained in Step 2**.

**Figure 4:**
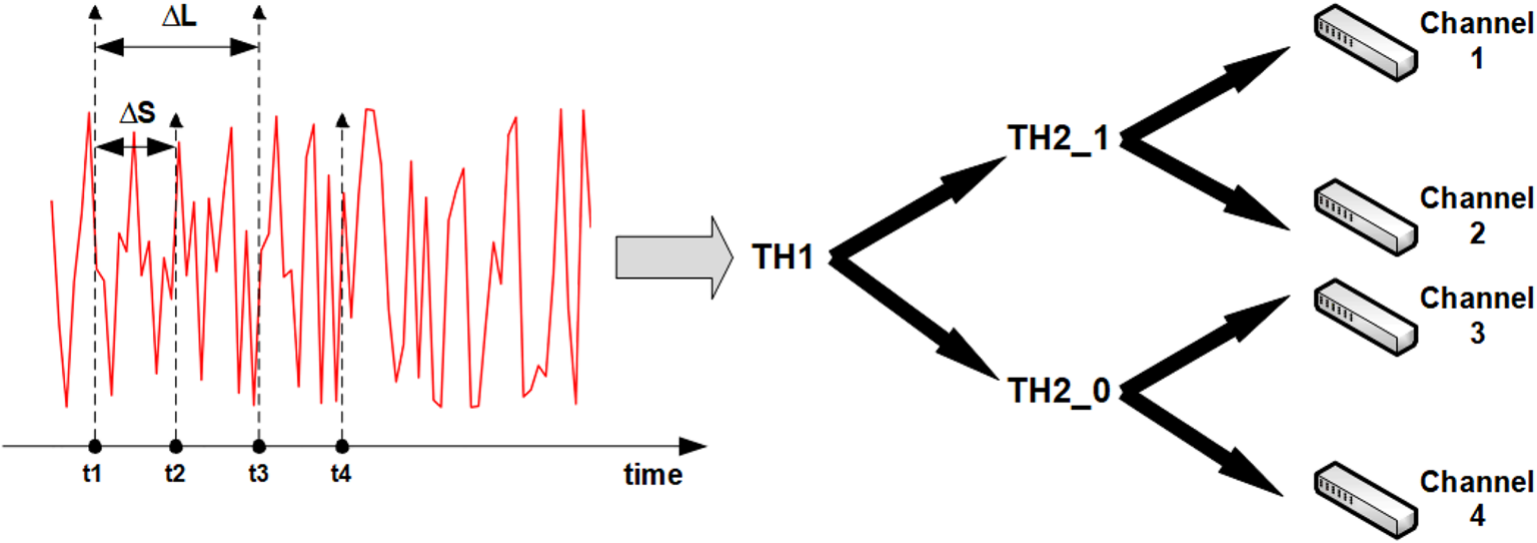
The process of using the laser chaos series to select the corresponding channel when *M*=2.

This last step is to adjust the threshold according to the input obtained in the previous step. If the return is greater than 0, the threshold is adjusted to the direction that $k'$ is more likely to be chosen. Otherwise, the threshold is adjusted to the direction where $k'$ is less likely to be chosen. The adjustment of the threshold is performed by adding and subtracting the two parameters $\Lambda$ and $\Omega$. The parameter which will be adjusted is determined by the size of the arm gain. If the arm gain is greater than 0, the threshold is adjusted using the parameter $\Lambda$ and vice versa. The parameters are divided into fixed and flexible types. The values of $\Lambda$ and $\Omega$ under the fixed type are both 1. For the flexible type, $\Lambda$ is set to be 1 and $\Omega$ is continuously adjusted according to the selection as follows^17^:

$$\begin{aligned} \Omega _{i,S_{1}S_{2}\ldots S_{i-1}} = \dfrac{\frac{L_{i,S_{1}S_{2}\ldots S_{i} =0}}{T_{i,S_{1}S_{2}\ldots S_{i} =0}}+\frac{L_{i,S_{1}S_{2}\ldots S_{i} =1}}{T_{i,S_{1}S_{2}\ldots S_{i} =1}}}{2-\Big (\frac{L_{i,S_{1}S_{2}\ldots S_{i} =0}}{T_{i,S_{1}S_{2}\ldots S_{i} =0}}+\frac{L_{i,S_{1}S_{2}\ldots S_{i} =1}}{T_{i,S_{1}S_{2}\ldots S_{i} =1}}\Big )},\ \ \ i=1,\ldots ,M, \end{aligned}$$

where $L_{i,S_{1}S_{2}\ldots S_{i}=j}$ indicates the selection times of observing positive return when $S_{1}S_{2}\ldots S_{i-1}$ have been determined with $S_{i} = j, j\in \{0,1\}$ and $T_{j,S_{1}S_{2}\ldots S_{i}=j}$ indicates the selection times when $S_{1}S_{2}\ldots S_{i-1}$ have been determined, and $S_{i}=j$. The two parameter types are suitable for different distribution situations; thus, an appropriate parameter type is chosen according to the channel distribution. In more detail, we present the process of using laser chaos series to select a channel with $M=2$ (Fig. 4).

### Parallel exchange process

**Figure 5:**
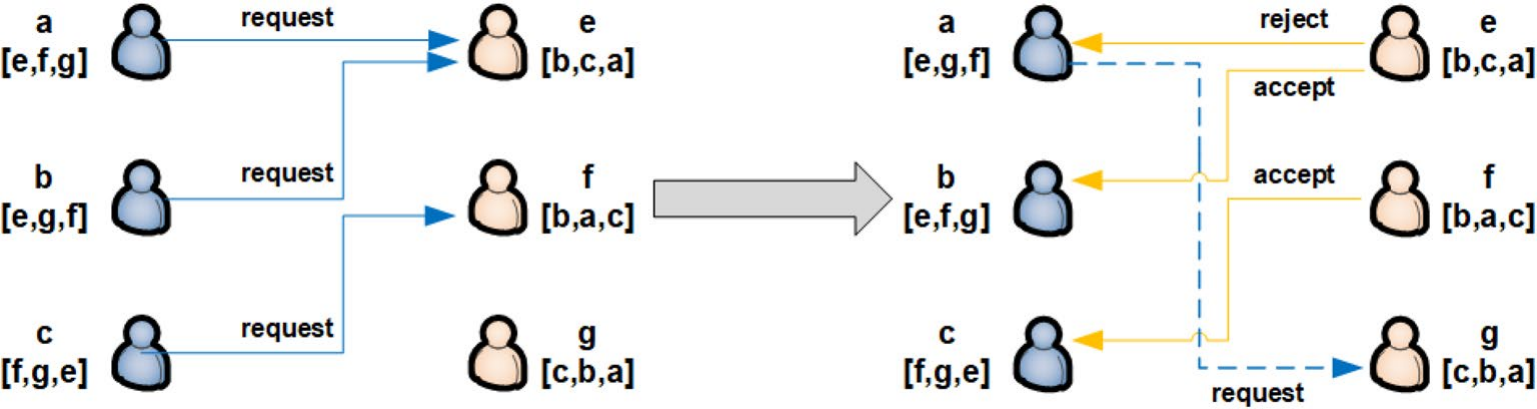
Users $\{a,b,c\}$ and users $\{e,f,g\}$ are paired based on the marriage matching model with their selection preference.

The basic thought of this exchange model is derived from the marriage matching model, which can be described by the following scenario (Fig. 5). Suppose that there are two sets *A*, *B*, each of which contains *L* individuals. Every person has his/her unique preferences. Considering $L=3$ as example, the sets of individuals are given by $A:=\{a,b,c\}$ and $B:=\{e,f,g\}$. The selection preference of individuals in *A* is given by the strictly ordered sequence [*f*, *e*, *g*], in which the level of attraction decreases constantly. At the beginning of the first round, the individuals in *A* sequentially select their preferred individual from *B*. If an individual in *B* is chosen by a single individual in *A*, the combination is tentatively established. If an individual in *B* is chosen by multiple individuals in *A*, the individual with higher priority, as determined by the preference of the individual in *B*, is selected. The remaining individuals in *A* proceed to the next round and repeat the process until each individual in *A* is paired with a single individual in *B*.

The multiuser channel selection model is similar to the marriage matching model. The individuals in *A* can be analogized to users, whereas the individuals in *B* can be likened to channels. The phenomenon that one person in *A* can only be paired with an unmatched person in *B* agrees with previous assumptions in dynamic channel selection issues. Therefore, this study uses this principle to handle each user’s selection. In addition, this paper proposes a parallel processing method where all users perform channel selection (stable marriage model) simultaneously. Compared with a single initiator at a given time, the simultaneous operation of multiple users intends to get more effective information throughput. The detailed parallel exchange process is discussed below.

**Figure 6:**
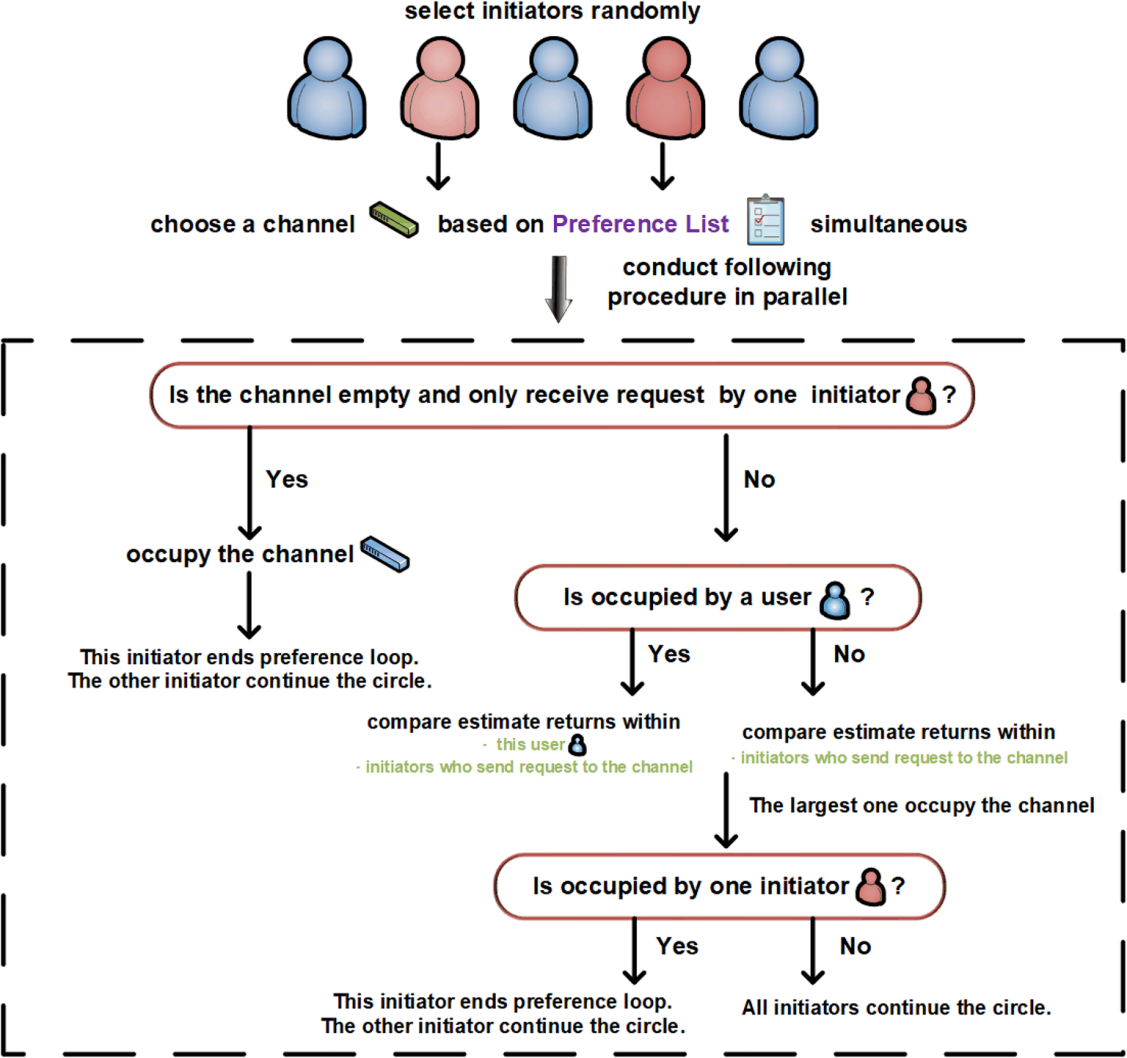
Exchange steps with SCA as the target state.

**Figure 7:**
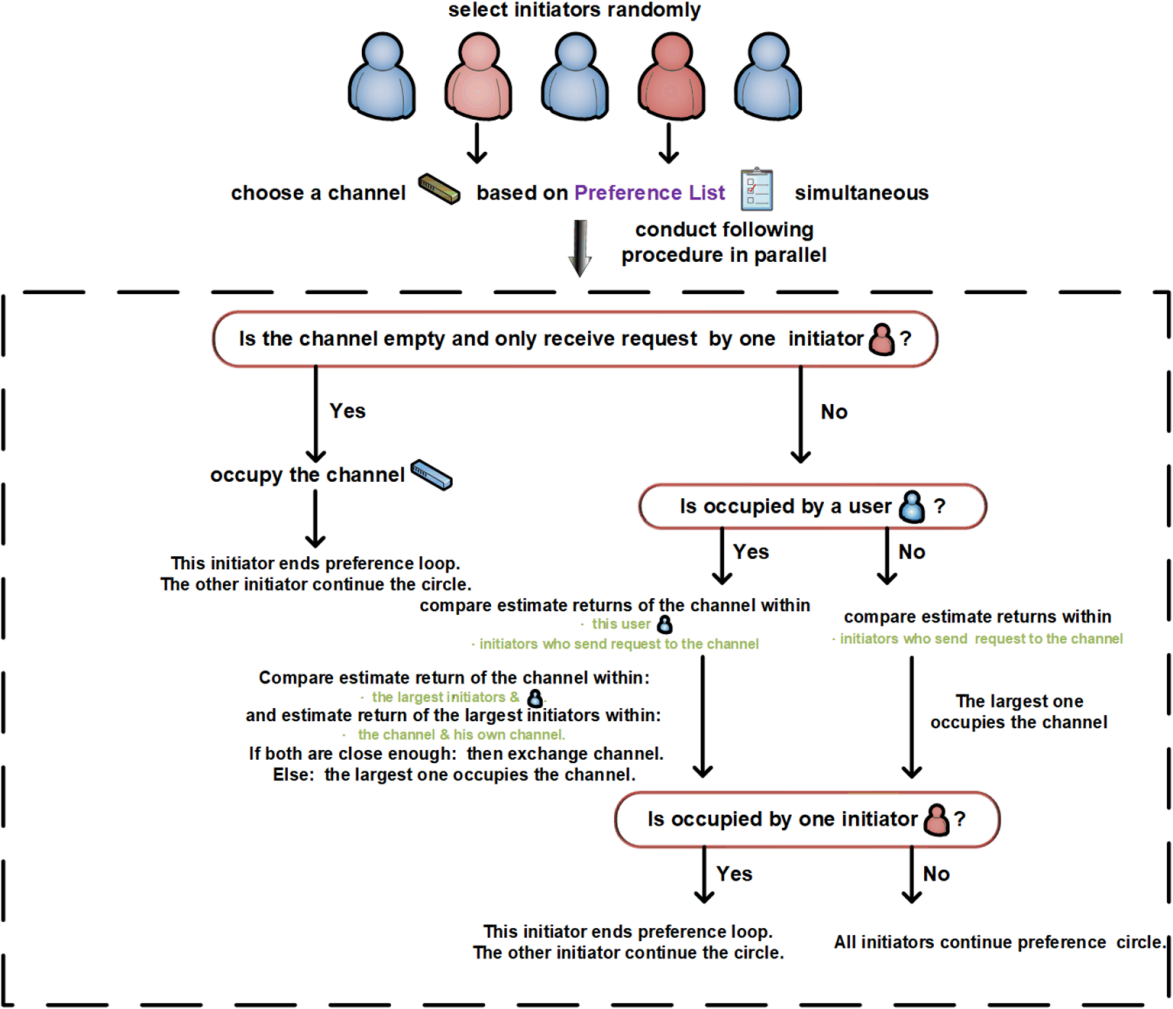
Exchange steps with FSCA as the target state.

The exchange phase at every time slot *t* is divided into two parts. First, initiators are selected randomly from users, which differs from the marriage matching model with only initiators exchanging channels, not all users. Second, the randomly selected initiators will launch requests to channels in parallel. Notably, the exchange steps are closely related to the target states. For the exchange step with SCA as the target state, the selected initiators send requests to channels cyclically according to their preferences. If a channel is empty and no other requests are received, the channel is occupied by the request sender. If channel $k_{1}$ receives multiple requests from initiators, compare all $\hat{\mu }_{t}^{init, k_{1}}$ (including $\hat{\mu }_{t}^{n_1, k_{1}}$ if user $n_1$ occupies channel $k_{1}$ at time slot *t*), where *init* indicates all initiators sending requests to channel $k_{1}$. The user with the largest value occupies channel $k_1$. In addition, if the largest one is the initiator, the channels are exchanged with user $n_1$ and the current preference cycle is terminated. Otherwise, the cycle continues. For the exchange step with FSCA as the target state, the process is similar to that of SCA, with the major difference being the exchange condition. The detailed steps for SCA and FSCA are shown in Fig.6 and 7, respectively.

## Results

### The performance of PPL-LC with SCA

**Figure 8:**
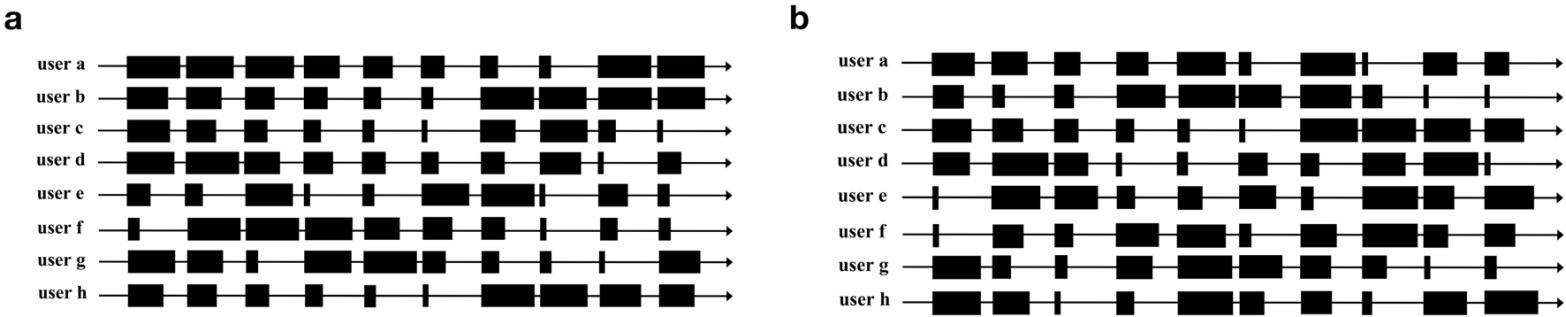
Initial channel distribution settings. (**a**) The distributions of users for each channel during 0–3000 iterations. (**b**) The distributions after 3000 iterations.

The PPL-LC algorithm is initially tested using SCA as the target state. Ten channels and eight users, designated as $\{a, b,\ldots ,h\}$, are configured. The initial and subsequent channel distributions of each user are set as follows (Fig. 8). The size of each black block represents the initial channel distribution set by each user. In other words, the larger the black block, the greater the user’s satisfaction with the channel and the higher the probability of successful data transmission. Notably, these data settings will be used throughout the experiments.

A small number of channel exchanges may prevent the algorithm from achieving the maximum information throughput. When there is a small number of channel exchanges, the information throughput may reach equilibrium more quickly, resulting in a local optimum and an inability to achieve the maximum information throughput. However, an excessive number of channel exchanges can also cause resource waste between users. Therefore, it is crucial to balance the relationship between the number of channel exchanges and throughput with two aspects. On the one hand, it is necessary to ensure sufficient exchange times so that the information throughput achieved by all users will not fall into the local optimum. On the other hand, it is also necessary to control the number of channel exchanges so as not to waste resources.

**Figure 9:**
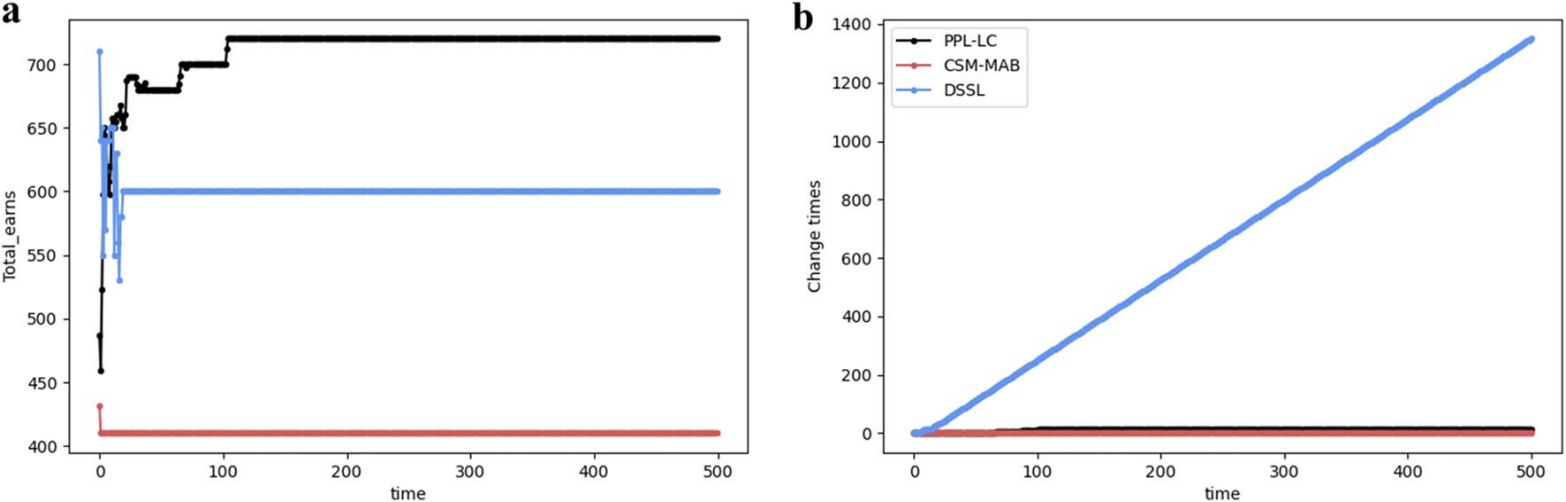
The performance comparison of the PPL-LC, DSSL, and CSM algorithms. (**a**) The trend of transmitted information bits over time. (**b**) The performance exchange times over time.

When the channel distribution does not change over time *t*, the relationship between the exchange amount and time, as well as the transmitted information bits and time, are examined separately. In Fig. 9, the performances of the PPL-LC, DSSL, and CSM-MAB algorithms with respect to the aforementioned metrics are compared. Fig. 9a shows that the DSSL and CSM-MAB algorithms reach equilibrium faster, but the information throughput under the SCA state is lower (namely, the SCA state is not reached). Although it takes the PPL-LC algorithm longer (around 100 time slots) to reach the SCA state, it ultimately achieves higher information throughput. From Fig. 9b, the number of channel exchanges for the CSM-MAB algorithm is extremely lower than that of the DSSL algorithm, close to the exchange times of the PPL-LC algorithm, which may contribute to its inability to reach the SCA state. In addition, the number of channel exchanges for the DSSL algorithm is significantly higher than those of the other two algorithms, resulting in inefficient resource utilization in practical implementation.

**Figure 10:**
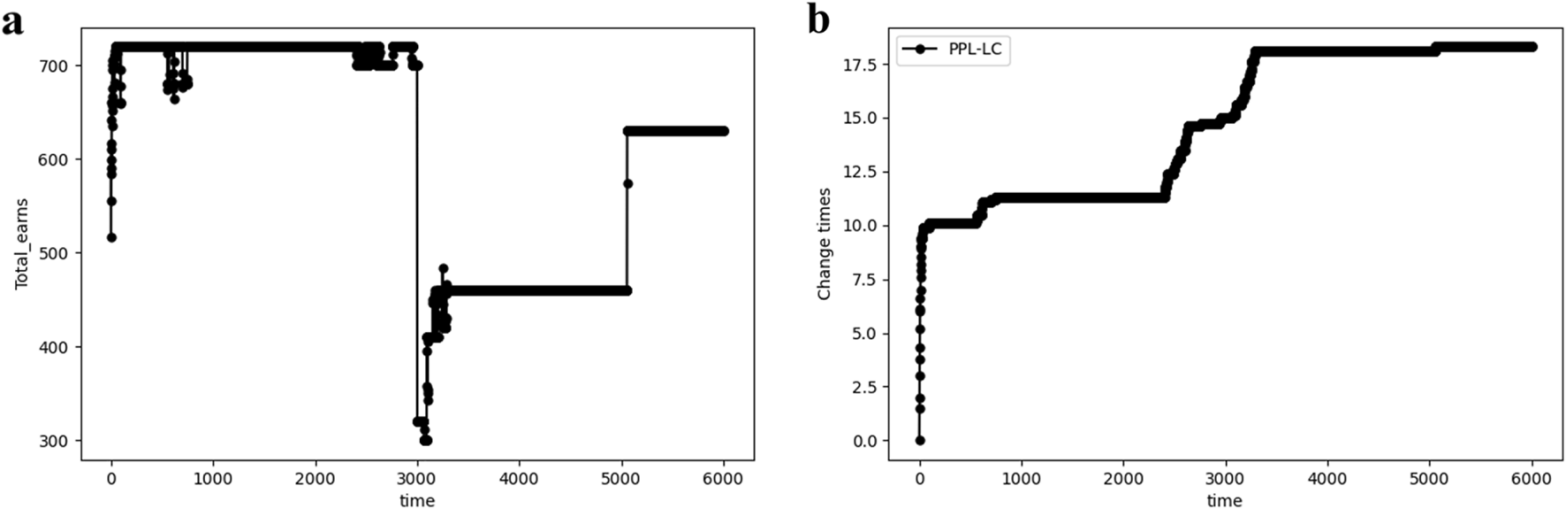
The performance of PPL-LC. (**a**) The relationship between transmitted information bits and time when distribution changes. (**b**) The relationship between the number of channel exchanges and the time when distribution changes.

After 3000 iterations, the channel distribution environment changes. As shown in Fig. 10, the SCA state has been achieved before the 3000th iteration. However, the information throughput experiences a sharp decline after the 3000th iteration. As a result, the channel exchange process is restarted, re-establishing of the SCA state under the new distribution. In conjunction with this, the number of channel exchanges, which had remained stable, also increased after the 3000th iteration.

### The performance of PPL-LC with FSCA

**Figure 11:**
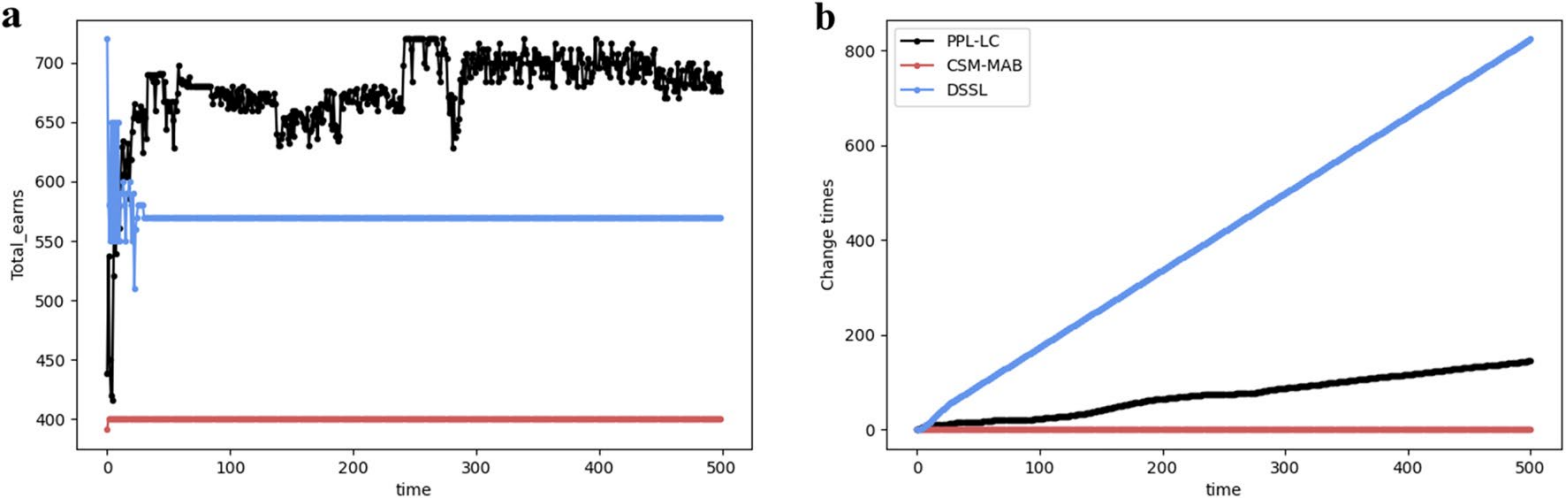
The performance of PPL-LC with FSCA. (**a**) The relationship between total information transmitted and time, which also exhibits the comparison with the DSSL and CSM algorithms. (**b**) The relationship between exchanges and time.

In the following, the FSCA state is considered the target state. Similar to the SCA state, the performances of the three algorithms in terms of throughput and change times are considered (Fig. 11). Under the FSCA target, the PPL-LC algorithm fluctuates after reaching a high information throughput, corresponding to the user’s overall selection switching back and forth between several fuzzy states. With more observations, the PPL-LC algorithm can result in the throughput tending to be stable. Meanwhile, the DSSL and CSM-MAB algorithms still cannot reach the FSCA state, and the number of channel exchanges of the CSM-MAB algorithm quickly reaches equilibrium, whereas the number of channel exchanges of the DSSL algorithm gradually increases as users continue to exchange between empty channels.

## Discussion

Previous research has demonstrated that laser chaos is effective at random number generation and can have a good effect on MAB problems. Considering these facts, the usefulness of ultrafast chaos sequences for real wireless applications was confirmed^14^. Despite this, there is inadequate research on the potential applications of the laser chaos series. In this study, we explored the high performance of ultrafast laser chaos for CRNs with multiple users. We proposed a parallel processing algorithm using laser chaos to attain two stable states: the classical SCA and FSCA. We discovered that the proposed algorithm provides improved performance, specifically in terms of throughput, in dynamic spectrum access. In addition, the algorithm demonstrates good adaptability to changing environments, which is a key feature in dynamic spectrum access scenarios where the channel conditions may vary over time. These combined capabilities make the proposed algorithm a valuable tool for improving the performance of CRNs with multiple users.

These findings of this study extend those of Takeuchi^14^ with multiple users and unlimited channel numbers, confirming that ultrafast laser chaos gives high performance in wireless communication networks. In addition, this study expands the classical SCA and describes the circumstance where users have vague preferences. Therefore, this study indicates that laser chaos has positive effects on wireless communication networks. Particularly, to the best of our knowledge, the proposed algorithm is the first to apply laser chaos to channel selection with multiple users. Although this study uses laser-chaos-based decision-making for dynamic channel selection, future work can focus on more general channel assignment scenarios.

## Methods

In this section, the proposed PPL-LC algorithm is presented, along with the parameter adjustment of the laser chaos algorithm.

### Brief description of the PPL-LC algorithm

**Figure 12:**
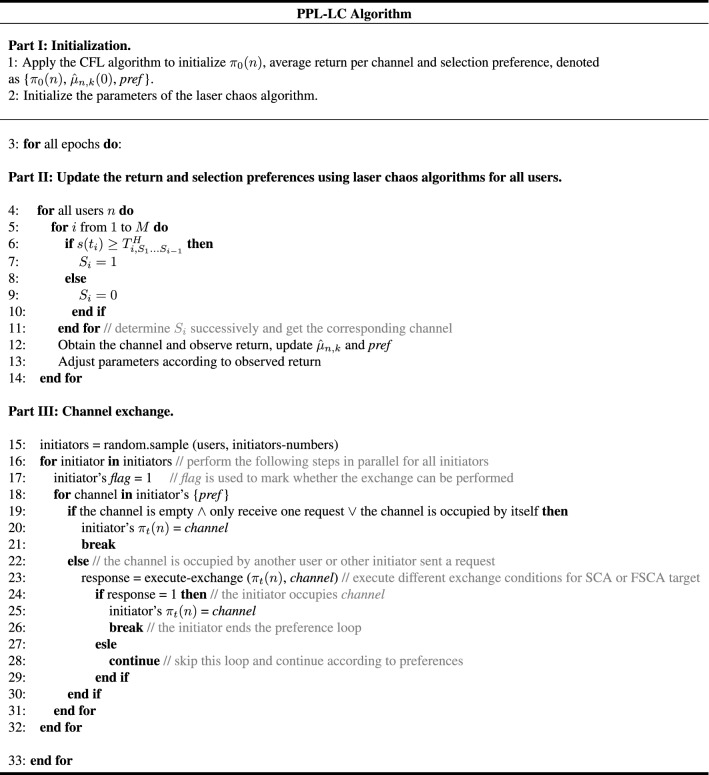
The PPL-LC algorithm.

With detailed discussions in previous sections, the PPL-LC algorithm is described in detail (Fig. 12). It comprises three parts: initializing, updating and exchanging. In the initializing part, it employs the Communication-free learning (CFL) algorithm^18^ to reach an initial orthogonal configuration (line 1). The CFL algorithm convergence quickly to an orthogonal configuration, allowing users to proceed with the updating and exchanging parts.

Next, in the updating part, users select the channel according to the random number generated by laser chaos (lines 5–10). Then,users send a message and obtain feedback. Based on this, the return and corresponding parameters are adjusted (lines 11–12). The above steps are all supported by the principle introduced in the laser chaos algorithm. Therefore, users obtain a list of channels they prefer over their current action.

Upon arriving at the exchanging part, randomly select initiators among users (line 15). All the initiators choose a channel based on their preference list simultaneously. Every initiator will then observe whether the channel is empty and only receiving one initiator’s request (line 19). If this occurs, he/she can occupy this channel immediately (lines 20–21). Otherwise, further observations are reqquired for the selection. The initiator proceeds to signal whether the desired channel is being occupied by a user who is not an initiator. If it is, this responding user will consider whether exchanging channels with the initiator will improve his/her osituation based on his/her preference ranking. If the responder agrees to the swap, it will be done (lines 24–26). If the responder refuses, the initiator will approach the next best channel on the list.

If the desired channel is not occupied by a user, but initiators, all the initiators who have sent requests to this channel will compare their estimated returns. The initiator with the largest estimated return will occupy the channel. If multiple initiators have the same highest estimated return, they will all move on to the next best channel on their list. All users will continue the process of seeking a partner who is willing to exchange channels with them until they either find one or exhaust their list of potential swaps. This part of the algorithm is illustrated in Fig. 10.

### Parameter adjustment

When user *n* occupies channel *k* to transmit information and receives a positive return, the parameters of the laser chaos algorithm are adjusted as follows:

$$\begin{aligned} T^H_{i,S_{1}\ldots S_{i-1}} = \alpha * T^H_{i,S_{1}\ldots S_{i-1}} + \Lambda (-1)^{S_{i}},\ \ i= 1,2,\ldots ,M-1,M. \end{aligned}$$

If the feedback return is equal to zero, it is updated with

$$\begin{aligned} T^H_{i,S_{1}\ldots S_{i-1}} = \alpha * T^H_{i,S_{1}\ldots S_{i-1}} + \Omega (-1)^{1-S_{i}},\ \ i=1,2,\ldots ,M-1,M. \end{aligned}$$

### Data analysis

For laser chaos generation, there are two ways to simulate: optical system and computer simulation. This study adopts computer simulation to obtain random numbers and the simulation with real optical systems will be studied in later papers. This simulation is mainly completed through the example-DiodeWithFeedbackSimulation in Matlab Simulate and the chaotic waveform is sampled to form a random sequence. Compared with building an optical system, this method is more convenient. The simulation is performed on a personal computer (R9000X 2021, R7 5800H 3.2GHz, 16GB RAM, Windows 11, Matlab R2016b).

### Parameters setting

In this study, the initial parameters of the laser chaos algorithm are selected as follows: $\Lambda = 1,\Omega = 1,\alpha = 0.99$. The values of $\Delta _{S}$ and $\Delta _{L}$ are the same as in the previous experiments, and we convert the signal level value obtained by laser chaos to 8 bits, namely, $-128 \le s(t) \le 127$^19^.

## Data Availability

The datasets generated during this study are available from the corresponding author upon reasonable request.
